# The perception of the leader as an attachment figure: can it mediate the relationship between work engagement and general/citizenship performance?

**DOI:** 10.1186/s40359-021-00700-9

**Published:** 2021-12-18

**Authors:** Elena Lisá, Katarína Greškovičová, Katarina Krizova

**Affiliations:** Faculty of Social and Economic Sciences, Comenius University in Bratislava, Mlynské luhy 4, 821 05 Bratislava, Slovakia

**Keywords:** Attachment figure, Leader, Follower, Work engagement, Work performance

## Abstract

The study aimed to explore the perception of the leader as a security provider as a potential mediator of the relationship between work engagement and perceived general and citizenship work performance. Five hundred and forty-two adults completed the Leader as a security provider scale, Utrecht work engagement scale, General work performance questionnaire, and Citizenship organizational behavior questionnaire to self-report on their organizational behaviors. The perception of the leader as a secure attachment figure partially mediated loyalty and adherence to the organization's rules in engaged employees. Perceived separation distress can increase interpersonal citizenship performance; however, it can decrease organizational compliance in engaged employees. Fear of losing the leader can potentially harm the organizational goals by favoring the personal relationships before organizational compliance.

## Introduction

The application of attachment theory in work and organizational settings has gained a lot of relevance in recent years [1–3]. Previous studies explored attachment in relation to organizational behaviors, such as job satisfaction, leadership, and trust [1] as well as organizational outcomes, such as job performance [2, 3]. Since job performance is critical to organizational effectiveness, applying attachment theory to explore factors contributing to enhanced job performance improvement might be particularly important. In the literature, job performance is typically linked with employee engagement [4]. Understanding employee engagement through the lens of employee attachment to their organizational leader, therefore, has a potential to explain the link between employee engagement and employee performance and expand on our current knowledge related to employee engagement and performance.

### Attachment models

According to attachment theory, a child has an innate need to attach to their primary caregiver [5]. Based on the interactions with their attachment figures, children develop attachment patterns that have been shown to manifest throughout their lives and across different relationships [6–8]. Additionally, the attachment patterns with their attachment figures also inform children’s internal working models—mental representations of themselves and other people with whom they form relationships. These internal working models affect behavior, thinking, and perception in all attachment relationships [7], and, contribute to the development of a global attachment model [9]. Global models represent the entire history of significant relationships and serve as default model guide for new relationships or novel situations [9]. Furthermore, the history of specific types of relationships (such as partnerships) are incorporated in domain-specific models, and the history of a relationship with a specific person (such as a current partner) in specific-relationship models [10]. The latter ones provide interpretation filters that affect behavior, cognition, and perception of the attached one in that specific relationship [11–13].

Global models are strongly related to overall psychological adjustment [12] and well-being [14], while specific models are related to relationship-specific outcomes, such relationship satisfaction [12, 14]. Since specific-relationship models serve as important interpretation filters of behaviors and perceptions of the other in specific attachment relationships, they seem particularly well-suited for exploration of workplace attachment relationships that significantly differ from other types of social relationships in boundaries, tasks, and goals [11–13]. In this study, we apply the specific-relationship attachment lens to explore the attachment between employees and their organizational leaders in order to better understand the link between employee engagement and employee performance.

### Organizational leader as an attachment figure

Even though that a feeling of security as the primary goal of attachment remains constant [5], other factors related to attachment, such as triggers of attachment behavior, ways of seeking proximity, even attachment figures themselves, might change [15]. People have multiple attachment figures as they go through life, for example their parents [5], romantic partners [16], and other significant adults [6]. In secure attachment pattern, attached people experience distress when separated from their attachment figures and attachment figures provide secure base (i.e., support, encouragement, responsiveness, and availability) and safe haven (i.e., protection, reassurance, soothing) when attached people seek proximity [5, 6, 17–19].

Hazan and Shaver [20] were the first to note that the functions of attachment figures can be observed in the workplace where leaders might serve as attachment figures for their employees. From an attachment perspective, leaders might serve as a secure base for their employees who turn to the leaders for support during stressful workplace situations, such as when a change or a loss occurs [3]. The understanding of leadership and employee behaviors through the attachment lens has recently gained a lot of attention [21–24]. Molero et al. [24] developed a questionnaire to examine the perception of the leader as a security provider and found that this perception is related to various organizational variables, such as transformational leadership, satisfaction with management, perception of the manager's efficiency, authentic leadership, organizational identification, work engagement, work satisfaction, and job burnout [24]. The perceived leaders’ support was also found to positively predict employees’ proactive behavior in a recent study [23]. Furthermore, the supervisors’ support, recognition, and feedback were found to be salient in developing and maintaining work engagement of newly employed workers [25]. Engagement and willingness to perform above expectations are the most critical employability skills for recent employers [26].

These studies’ findings indicate that employee-leader relationship research can be expanded to and benefit from an attachment perspective. Since organizations should strive to support employee productivity by utilizing empirically confirmed predictors of focal and contextual performance [4], the perception of a leader as an attachment figure might significantly contribute to our knowledge of employee performance, and consequently, enhance work outcomes.

### Work engagement, performance and leaders as attachment figures

While the positive connection between the perception of a leader as an attachment figure and work engagement is hypothesized [25], the research in this area is novel and therefore, only a handful of previous studies examined this connection within an organizational environment [23–25, 27]. In their seminal work describing the development of the perception of the leader as a security provider scale, Molero et al. [24] found a positive association between the leader security perception and work engagement. Similarly, a different study found that the perceived leaders’ support predicted employees’ proactive behavior [23]. Stable relational models of attachment might, therefore, either facilitate or constrain employees’ engagement [27].

Engaged employees contribute to organizational performance and contextual performance by creating a social context that enhances organizational effectiveness [28]. Engagement is considered to be a significant predictor of employee effectiveness for both focal and contextual work performance [4]. Work performance is usually described as direct and indirect contributions of an individual to an organization's goals [29]. It consists of two interplaying components: task performance and contextual performance. Task performance refers to in-role or formal job performance, and can be described as the expertise with which employees perform basic technical activities relevant to the job. In this study, task performance was operationalized as the general performance. Contextual performance is characterized by activities that contribute to the psycho-social benefit of the organization [30]. These activities may not be registered or paid [29, 31], but are very welcome by employers [26]. Contextual performance is defined similarly to citizenship organizational behavior as “individual behavior that is discretionary, not directly recognized by the formal reward system, and that in the aggregate promotes the effective functioning of the organization” (p. 86) [30].

Coleman and Borman [32] defined a 3-cluster data-driven model of citizenship performance. According to these authors, citizenship performance has three components (p. 36): (1) Interpersonal Citizenship Performance defined by behaviors that assist, support, and develop organization members through cooperative and facilitative efforts that go beyond expectations; (2) Organizational Citizenship Performance defined by citizenship behaviors that demonstrate commitment to the organization through allegiance and loyalty to the organization and organization objectives, and compliance with organizational rules, policies, and procedures; and (3) Job/Task Conscientiousness defined by extra efforts that go beyond role requirements and that demonstrate dedication to the job, persistence, and the desire to maximize one's own job performance.

### Goal of the current study

Several studies consider leaders as important participants in attachment research [15, 21, 22, 24], but there are only a few studies that focused on the perception of the leader as an attachment figure [23, 24]. Despite the pioneering work of Hazan and Shaver [20], leaders are usually not acknowledged as attachment figures. Work engagement as a confirmed predictor of complex organizational performance is expected to be enhanced by a supportive organizational environment and a culture, that can be fostered by a perception of the leader as a security provider [4]. Studies focused on the role of attachment in predicting work outcomes, typically examine attachment in workplace as a mediating or a moderating factor [1, 2]. No previous studies examined the perception of the leader as an attachment figure as a potential mediating variable between employee engagement and organizational outcomes. Moreover, recent studies focus on attachment style as a global or domain model of attachment and lack in considering the specificity of work relationships in between employees and their leaders. Therefore, the current study focused on the perception of the leader as an attachment figure as a mediating variable between work engagement and work performance. We stated the following hypotheses:

#### **H1**

The perception of the leader as a security provider has a mediating effect on the relationship between work engagement and general work performance.

#### **H2**

The perception of the leader as a security provider has a mediating effect on the relationship between work engagement and citizenship performance.

## Materials and methods

### Participants

Participation in the research was voluntary and anonymous. Participant recruitment and the questionnaire’s administration in a paper format were conducted by University students enrolled in the “Psychological assessment” course as a part of their final grade. The participants provided their written informed consent. They agreed with the study’s use of their aggregated anonymous data for research purposes. All procedures performed in our study followed the ethical standards of the 1964 Helsinki declaration and its later amendments and the Internal Institutional Regulation 5/2014. A local ethics committee FSES CU upon the Regulation 5/2014 ruled that no formal ethics approval was required in this particular case.

The sample consisted of 542 Caucasian employees (42% men, 52% women, 6% chose not to answer), between ages 16 and 78 (*M* = 37.8; *SD* = 11.3). The years during which our participants worked with their current organizational leader ranged from 0 to 40 years (*M* = 5, *SD* = 5). Sixty-three percent of participants did not share their work area, and the rest of the sample worked in various market sectors (finance, education, sale, administration).

### Measures

*The leader as security provider scale (LSPS)* measures the perception of the leader as an attachment figure [24]. The original scale is one-dimensional and consists of 15 items that are based on five functions of a perceived attachment figure as outlined by the attachment theory: secure base, safe haven, proximity seeking, emotional ties, and separation distress [24]. The participants rate the items on a 5-point Likert scale ranging from *0—strongly disagree* to *4—strongly agree*.

The scale was translated into Slovak language and its psychometric properties were tested. The original one-dimensional model did not show a good fit with the data gathered from our participants. (Table 1). Instead, exploratory factor analysis (rotation Promax) indicated a 2-factor model through parallel analysis. The two factors explained 57.8% of the variance. Factor 1, "Secure figure" (LSPS1) explained 36% of the total variance and it comprised of seven items from the original version (“I can count on my leader to support me when I propose new ideas or procedures.” “I think my leader would support my growth and advancement on the job.” “I trust that my leader will be pleased with and proud of my work.” “When I am under stress at work, my leader helps me to remain calm.” “I can count on my leader to be there for me, no matter what.” “If I need reassurance or encouragement, I can count on my leader to supply it.” “I can count my leader will support my efforts on the job.”). The internal consistency of this dimension was good (α = .886). LSPS1 describes an attachment figure that provides a secure base and a safe haven, and that responds to the employees' proximity seeking. Employees tend to perceive their attachment figure/leader as supportive and encouraging of their pursuits of non-attachment-related goals in a safe environment and they tend to perceive their leader as an available source of protection, comfort, calm, and reassurance in times of need [24]. Three items were loaded on the second factor that represented "Separation distress" (LSPS2; “If my leader moved to another organization or another position in this organization, I would try to go with him/her.” “I feel emotionally connected to my leader, whether our relationship is positive, negative, or a combination of the two.” “I don't let too much time pass without being in close contact with my leader.”). LSPS 2 explained 21.8% of the total variance. The internal consistency of the second dimension was good as well (α = .798). LSPS2 describes employees who build emotional ties with their leaders and who feel distressed when separated from their leaders.

**Table 1:** Confirmatory analyses

Model	*Χ*^2^	*df*	*p*	RMSEA	SRMR	CFI	TLI
Single factor model [24]	368.021	90	< .001	.110	.066	.858	.835
Two-factor model (LSPS1, LSPS2)	93.181	34	< .001	.082	.046	.950	.934

The two-factor model that was identified in our data showed an acceptable data fit (*X*^2^ = 93.181, *df* = 34, *p* < .001; TLI = .934, CFI = .950, RMSEA = .082 and SRMR = .046). Table 1 shows that the two-factor reduced model showed better fit with the gathered data than the original model.

*Utrecht Work Engagement Scale (UWES)* was created by Schaufeli and Bakker [33]. Participants were administered a short 9-item version of the questionnaire and asked to rate their engagement at work on a 7-point Likert scale (*0* = *never*; *6* = *always*, *every day*). An example item includes “At my work, I feel bursting with energy”. The internal consistency of this scale in our data was excellent (α = .931).

*General Work Performance Questionnaire (GP)* [31] measures an overall employee performance by a three-item questionnaire. Respondents rated their responses on a 7-point Likert scale, where 7 indicated high performance and 1 indicated low performance. Total score was calculated by averaging of the three items. The internal consistency was good (α = .795).

*Citizenship Organizational Behavior* (COB) [32] measures citizenship work performance. The scale consists of 27 items scored on a 5-point Likert scale (*1* = *never* to *5* = *always*). The scale includes three subscales: (1) interpersonal citizenship performance (altruism and conscientiousness), (2) organizational citizenship performance (allegiance/loyalty, compliance), and (3) job/task conscientiousness.

The scale was translated into Slovak language and its psychometric properties were tested. The original 3-cluster model did not fit our data well (Table 2). Exploratory factor analysis with rotation Varimax confirmed a 3-factor solution with a reduced number of items. Factor 1 (COB1) Organizational Allegiance/Loyalty (α = .862; 19.8% of the total variance) included items: (1) endorsing, supporting, or defending organizational objectives; (2) demonstrating conscientiousness in the support of the organization; (3) maintaining a positive attitude about the organization; and (4) promoting and defending the organization. Factor 2 (COB2) Interpersonal Citizenship Performance (α = .773; explained 19.7% of the total variance) included items: (1) engaging responsibly in meetings and group activities; (2) engaging in self-development to improve one's own effectiveness; (3) engaging the behavior that benefits individuals in the organization; (4) assisting co-workers with personal matters; 5) providing extra service or help to customers; and 6) suggesting procedural, administrative, or organizational improvements. Finally, factor 3 (COB3) Organizational compliance (α = .704; explained 12.3% of the total variance) consisted of items: (1) following organization rules and procedures; and (2) participating responsibly in the organization.

**Table 2:** Confirmatory analyses

Model	*Χ*^2^	*df*	*p*	RMSEA	SRMR	CFI	TLI
Three-cluster model [32]	1105.500	321	< .001	.093	.076	.771	.749
Reduced three-factor model (COB1, COB2, COB3)	131.601	51	< .001	.073	.054	.933	.914

Confirmatory factor analysis showed acceptable data fit of the reduced model (*X*^2^ = 131.601, *df* = 51, *p* < .001; TLI = .914, CFI = .933, RMSEA = .073, and SRMR = .054). Our reduced three-factor model showed better fit with our gathered data than the original model (Table 2).

### Procedures

The leader as a security provider scale (LSPS) [24] and Citizenship Organizational Behavior scale (COB) [32] were translated into Slovak language by two independent translators [34]. In order the determine the internal structure and data fit of the translated versions, the total sample of 542 participants was divided into two subsamples. Exploratory factor analysis (EFA) was utilized in the first subsample (N = 270) and confirmatory factor analysis was used in the second subsample (N = 272). When conducing EFA, we reduced the item if it did not saturate any of the factors or, conversely, if it saturated more than one factor. Several criteria of the model fit were assessed in the CFA [35, 36]: robust Standardized Root Mean Square Residual (SRMR; cut-off score 0.05 or lower), Tucker-Lewis Index (TLI; cut-off score: 0.95 or higher), the robust comparative fit index (CFI; cut-off score: 0.95 or higher), and Mean Square Error of Approximation (RMSEA; cut-off score: .08 or lower). As shown in Table 3, the results indicated that the proposed seven-factor model fit the data better than other alternative models (*X*^2^ = 1370.486; *p* < .001; RMSEA = .060; SRMR = .047; TLI = .892; CFI = .903). We concluded that the scales were measuring distinct constructs. The sequence of merged dimensions in models shows the natural proximity of COB, LSPS, or performance dimensions.

**Table 3:** Confirmatory factor analyses

Model	*Χ*^2^	*df*	*p*	RMSEA	SRMR	CFI	TLI
Seven-factor model (UWES, LSPS1, LSPS2, GP, COB1, COB2, COB3)	1370.486	506	< .001	.060	.047	.903	.892
The best six-factor model (COB1 + COB3, UWES, LSPS1, LSPS2, GP, COB2)	1481.269	512	< .001	.063	.049	.891	.880
The best five-factor model (COB1 + COB3 + COB2, UWES, LSPS1, LSPS2, GP)	1705.253	517	< .001	.069	.056	.866	.855
The best four-factor model (COB1 + COB3 + COB2, UWES, LSPS1 + LSPS2, GP)	1928.409	521	< .001	.075	.060	.841	.829
The best three-factor model (COB1 + COB3 + COB2 + GP, UWES, LSPS1 + LSPS2)	2315.195	524	< .001	.085	.068	.798	.784
The best two-factor model (COB1 + COB3 + COB2 + GP + UWES, LSPS1 + LSPS2)	2925.243	526	< .001	.098	.081	.730	.712
Single-factor model (COB1 + COB3 + COB2 + GP + UWES + LSPS1 + LSPS2)	4303.905	527	< .001	.122	.113	.575	.547

*UWES* work engagement, *LSPS1* secure figure, *LSPS2* separation distress, *GP* general work performance, *COB1* organizational allegiance/loyalty, *COB2* interpersonal citizenship performance, *COB3* organizational compliance

The data were analyzed using the IBM SPSS Statistics 20 and JASP 0.14.1.0 software. Descriptive analysis, Pearson correlation analysis, exploratory factor analysis, confirmatory factor analysis, *t*-tests, and mediation analysis with bootstrapping were used to analyze the data.

## Results

Descriptive statistics and correlations between variables can be found in Table 4. Age and number of years with the current leader relate to the variables with small effect size. There was a statistically significant difference between women and men in COB, but with a small effect size (d = − .293). We conclude that the demographics have no significant impact on measured variables. These findings agree with no differences in demographics for perceiving a leader as a security provider [24] or employee engagement [37].

**Table 4:** Means, standard deviations, and correlations between variables

Variable	1		2		3		4		5		6		7		8		9
1. Age	–																
2. TWL	0.453	***	–														
3. UWES	0.173	***	0.175	***	–												
4. GP	0.218	***	0.177	***	0.370	***	–										
5. LSPS1	-0.025		0.020		0.404	***	0.116	**	–								
6. LSPS2	-0.036		0.079		0.338	***	0.128	**	0.550	***	–						
7. COB1	0.215	***	0.096	*	0.634	***	0.355	***	0.381	***	0.266	***	–				
8. COB2	0.124	**	0.126	**	0.503	***	0.379	***	0.309	***	0.332	***	0.531	***	–		
9. COB3	0.166	***	0.065		0.368	***	0.307	***	0.264	***	0.042		0.517	***	0.350	***	–
Mean	37.8		5		4.948		5.427		2.602		1.558		3.956		3.472		4.450
SD	11.3		5		1.085		0.804		0.722		0.912		0.772		0.700		0.648

*Age* age in years, *TWL* time with the current leader in years, *UWES* work engagement, *LSPS1* secure figure, LSPS2: separation distress, *GP* general work performance, *COB1* organizational allegiance/loyalty, *COB2* interpersonal citizenship performance, *COB3* organizational compliance

**Fig. 1:**
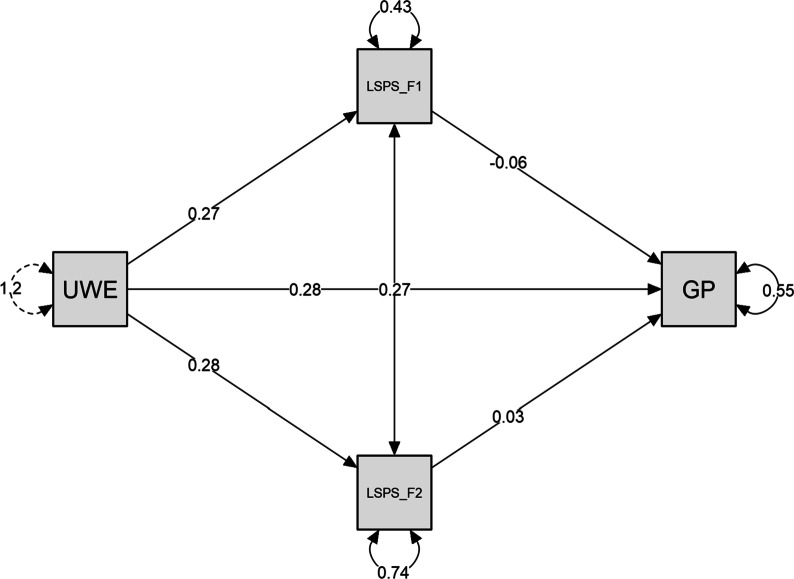
Path plot. *Note:* UWE: work engagement; LSPS_F1: secure figure; LSPS_F2: separation distress; GP: general work performance

To test Hypothesis 1, we included general work performance as a dependent variable, work engagement as a predictor, and two dimensions of the perception of the leader as a security provider construct (LSPS1: secure figure, LSPS2: separation distress) separately as two mediating variables in our first model (Fig. 1).The results in Table 5 show that the indirect effect of work engagement on general work performance through the perception of the leader as a security provider was not significant (Estimate of total indirect effect = − .008, SE = .014, z = − 0.547, *p* = .584). The direct effect of work engagement on general work performance was significant (Estimate of direct effect = .281, SE = .031, z = 9.143, *p* < .001), indicating that the perception of the leader as a security provider has no effect on general work performance as a mediator, nor any of its dimensions. Thus, the results do not support Hypothesis 1.

**Table 5:** Mediation analysis of the path from work engagement to general work performance mediated through secure figure and separation distress

		Std. error	z-value	*p*	95% confidence interval	
					Estimate	Upper
*Direct effects*						
UWES → GP	0.281	0.031	9.143	< .001	0.210	0.357
*Indirect effects*						
UWES → LSPS_F1 → GP	− 0.016	0.015	− 1.044	0.297	− 0.048	0.021
UWES → LSPS_F2 → GP	0.008	0.012	0.653	0.514	-0.020	0.031
*Total effects*						
UWES → GP	0.273	0.027	9.945	< .001	0.211	0.340
*Total indirect effects*						
UWES → GP	− 0.008	0.014	− 0.547	0.584	− 0.034	0.028

Delta method standard errors, bias-corrected percentile bootstrap confidence intervals, ML estimator

*UWES* work engagement, *LSPS1* secure figure, *LSPS2* separation distress, *GP* general work performance

**Fig. 2:**
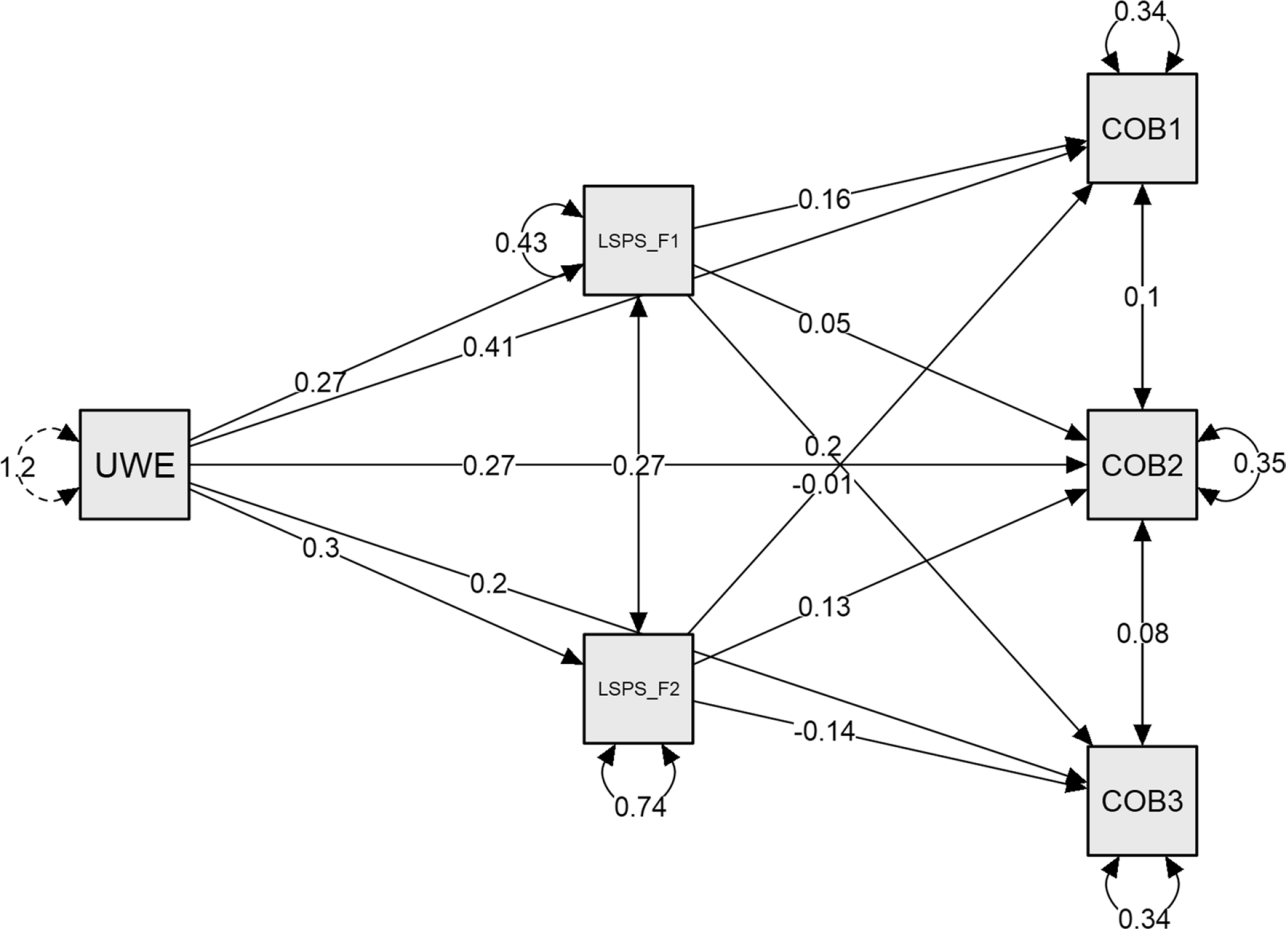
Path plot. *Note:* UWE: work engagement; LSPS_F1: secure figure; LSPS_F2: separation distress; COB1: organizational allegiance/loyalty; COB2: interpersonal citizenship performance; COB3: organizational compliance

To test Hypothesis 2, the analyzed model (Fig. 2) included the three dimensions of the citizenship performance construct (COB: 1 organizational allegiance/loyalty, COB2: interpersonal citizenship performance; COB3: organizational compliance) as dependent variables, work engagement as a predictor, and two dimensions of the perception of the leader as a security provider construct (LSPS1: secure figure, LSPS2: separation distress) as two mediators.

The results in Table 6 show that the indirect effect of work engagement on organizational allegiance/loyalty through the perception of the leader as a security provider was significant (Estimate of total indirect effect = .042, SE = .012, z = 3.587, *p* < .001). Specifically, the dimension of the secure figure was a significant mediator (Estimate of indirect effect = .045, SE = .012, z = 3.590, *p* < .001); however, the dimension of separation distress was not. Additionally, the direct effect of work engagement on organizational allegiance/loyalty was also significant (Estimate of direct effect = 0.409, SE = .024, z = 16.833, *p* < .001), indicating that the secure figure dimension is a partial mediator (10%) of the relationship between work engagement and organizational allegiance/loyalty.

**Table 6:** Mediation analysis of the path from work engagement to citizenship work performance mediated through secure figure and separation distress

	Estimate	Std. error	z-value	*p*	95% confidence interval	
					Lower	Upper
*Direct effects*						
UWES → COB1	0.409	0.024	16.833	< .001	0.348	0.467
UWES → COB2	0.272	0.025	11.069	< .001	0.218	0.328
UWES → COB3	0.205	0.024	8.457	< .001	0.155	0.257
*Indirect effects*						
UWES → LSPS_F1 → COB1	0.045	0.012	3.590	< .001	0.018	0.078
UWES → LSPS_F2 → COB1	− 0.002	0.010	− 0.241	0.809	− 0.022	0.017
UWES → LSPS_F1 → COB2	0.014	0.012	1.190	0.234	− 0.011	0.044
UWES → LSPS_F2 → COB2	0.038	0.011	3.524	< .001	0.019	0.064
UWES → LSPS_F1 → COB3	0.055	0.013	4.377	< .001	0.030	0.087
UWES → LSPS_F2 → COB3	− 0.041	0.011	− 3.803	< .001	− 0.066	− 0.019
*Total effects*						
UWES → COB1	0.451	0.022	20.566	< .001	0.399	0.497
UWES → COB2	0.324	0.022	14.594	< .001	0.281	0.374
UWES → COB3	0.219	0.022	9.925	< .001	0.175	0.265
*Total indirect effects*						
UWES → COB1	0.042	0.012	3.587	< .001	0.019	0.070
UWES → COB2	0.053	0.012	4.223	< .001	0.027	0.084
UWES → COB3	0.014	0.012	1.192	0.233	− 0.011	0.042

Delta method standard errors, bias-corrected percentile bootstrap confidence intervals, ML estimator

*UWES* work engagement, *LSPS1* secure figure, *LSPS2* separation distress, *COB1* organizational allegiance/loyalty, *COB2* interpersonal citizenship performance, *COB3* organizational compliance

The indirect effect of work engagement on interpersonal citizenship through the perception of the leader as a security provider was significant (Estimate of total indirect effect = .053, SE = .012, z = 4.223, *p* < .001). Specifically, separation distress was a significant mediator (Estimate of indirect effect = .038, SE = .011, z = 3.524, *p* < .001), but secure figure was not. Additionally, the direct effect of work engagement on interpersonal citizenship was also significant (Estimate of direct effect = .272, SE = .025, z = 11.069, *p* < .001), indicating that separation distress is a partial mediator (12%) in the relationship between work engagement and interpersonal citizenship.

The indirect effect of work engagement on organizational compliance through the perception of the leader as a security provider was not significant (Estimate of total indirect effect = .014, SE = .012, z = 1.192, *p* = .233). However, secure figure was a significant positive (Estimate of indirect effect = .055, SE = .013, z = 4.377, *p* < .001), but separation distress was a significant negative mediator (Estimate of indirect effect = − .041, SE = .011, z = − 3.803, *p* < 0.001). Additionally, the direct effect of work engagement on organizational compliance was significant (Estimate of direct effect = 0.205, SE = 0.024, z = 8.457, *p* < 0.001), indicating that secure figure (25%) and separation distress (19%) each played a partial mediating role in the relationship between work engagement and organizational compliance. The results partially support Hypothesis 2. The perception of the leader as a security provider has a mediating effect on the relationship between work engagement and organizational compliance. Perceiving leader as a secure figure significantly mediates the relationship between work engagement and organizational allegiance/loyalty, however; not between work engagement and interpersonal citizenship performance. On the other hand, the perception of separation distress from leader significantly mediates the relationship between work engagement and interpersonal citizenship performance, but not organizational allegiance/loyalty.

## Discussion

This is a novel study in its examination of the perception of the leader as a security provider variable’s mediating effects on the relationship between employee work engagement and work performance. We applied the relationship-specific attachment lens [9] to understand leaders as security provides in organizational settings to better understand relationship-specific outcomes [10].

First, we examined the mediating effects of the leader as a security provider variable on relationship between work engagement and general work performance. The leader as a security provider variable statistically significantly correlated with general work performance; however, its mediating effect on general work performance in engaged employees was not supported (H1). Previous research reported mixed results about performance and attachment styles: some studies found no relationship between attachment styles and job performance [38], while others found positive relationship between secure attachment style and job performance [39]. As Harms [1] stated, performance outcome is not necessarily always interpersonal, thus, the potential connection to attachment might be missing. It seems that the perception of the leader as a security provider is very personal in its nature and, therefore, does not relate to general work performance. Hence, we question whether the perception of the leader as an attachment figure is essential for employees' performance.

Testing of our second hypothesis revealed that the perception of the leader as a secure figure mediated the relationship between work engagement and organizational allegiance/loyalty and organizational compliance. Engaged workers that perceived their leader as a secure figure were keen to follow the rules and procedures and participate responsibly in the organization. At the same time, they support or defend organizational objectives, have a positive attitude about the organization, and promote or defend the organization. However, separation distress mediated organizational non-compliance in engaged workers. Engaged workers who feel separation distress are prone to avoid rules and responsible participation. However, they cooperate in meetings and group activities, help co-workers or customers, and concentrate on themselves and their benefits. The leader's support when perceived as an attachment figure was previously found to positively predict employees' proactive work behaviors, including increased positive emotions at work, and diminished feelings of job burnout, such as emotional exhaustion, cynicism, and incompetence [23]. Separation distress also mediated the prediction of interpersonal citizenship behaviors. Our findings show that the separation distress contributes to relationships with co-workers, interest in customers, and self-development.

Our findings only partially support the importance of the leader as an attachment figure when it comes to work performance. The leader-employee relationship is vastly different from parent–child or partner relationships, and it also has different objectives, therefore, the application of attachment theory is less straightforward. The leader-employee relationship does not need to include emotional closeness/ties in such intensity as child/parent or a romantic partnership attachment does. Attachment to leader is categorized under other domain and specific relationship models that include a history of relationships with previous leaders and the current leader [10]. From an attachment perspective, the construct of support is described by two concepts: a secure base meaning that one feels secure enough to go and explore (or work) and a safe haven meaning that one can approach the leader to find comfort when needed. These conceptualizations are partially incorporated in other concepts, such as psychological safety [40], inclusive leadership [41], or transformational leadership that are associated with the perception of the leader as an attachment figure concept [24].

Our results suggest that leaders might not be fulfilling all five functions typical for attachment figures [5, 6, 17–19]. After all, attachment figures, leaders included, are not expected to meet all the needs of the attached person at all times [18]. Other needs in the attachment relationship could be handled by another person in the workplace [19, 42] who might be involved in the attachment network of the employee [43]. Furthermore, the attachment figures in the workplace do not replace the attachment figures in everyday life but could instead serve as supplements to more important relationships that exist in employees’ personal lives. People form new relations to attachment figures during the lifespan, and the relevance of these attachments also vary depending on the situation [43]. An employee might see the leader as an attachment figure to a certain degree, but they might fear losing the leader if the relationship is too personal. The perception of the leader as a security-providing figure can contribute to loyalty and adherence to the organization's rules in engaged employees. The fear of being separated from the leader in engaged employees can potentially harm the organization, such as ignoring the rules, higher focus on relationships, and neglecting the organizations' goals. The leader should provide support with a professional distance.

Although previous literature notes that attachment is stable over one's life [44–48], it is possible for the leaders to foster their attachment ties with their employees by providing them with mentoring/coaching [2]. The attachment stability in relationships with new partners was found to be less stable compared to stability of established and long-term relationships [49]. Moreover, attachment can be influenced by environmental factors [50], and it is possible to create a sense of security by priming [51]. A leader perceived as a secure attachment figure might also help foster other positive aspects of an employee’s life, such as a sense of meaning [52], an experience of positive emotions [53], and resilience [54].

### Limitations

This study has several limitations. First, we used only self-report measures and we could not compare self-perceived performance scores to any objective indicators of work performance [55].

We shortened two questionnaires in order to increase our model fit. Therefore, some doubts could arise about the shortened versions’ validity. However, our results supported a positive relationship between secure attachment, engagement, and organizational citizenship behavior [56]. Moreover, a secure attachment provides flexibility from a cultural perspective and is not typical for any culture, whereas insecure attachments provide a good fit to specific cultural contexts [57].

The individual attachment model might determine how the employee perceives, interprets, and responds to their leader as a security provider, which might guide the employee’s workplace behavior more than the leader’s actual behavior [58]. Future research might focus on how the adult attachment style is related to the leader's perception as a safe figure and how this relationship predicts the employee's work performance.

## Conclusion

Our study brings new information about the perception of the leader as an attachment figure and its mediating effects on the relationship between employee work engagement and work performance. The perception of the leader as a security provider was found to partially mediate the organizational allegiance/loyalty and organizational compliance in engaged employees. On the other hand, perceived separation distress can increase interpersonal citizenship performance and decrease organizational compliance in engaged employees. Results suggest that a relationship with a leader that evokes the fear of losing the leader can harm the organizational goals by favoring the personal relationships before organizational compliance.

## Data Availability

The datasets generated during and/or analyzed during the current study are available from the corresponding author on reasonable request.
